# Critical Role of Toll-Like Receptor 9 in Morphine and *Mycobacterium tuberculosis*–Induced Apoptosis in Mice

**DOI:** 10.1371/journal.pone.0009205

**Published:** 2010-02-19

**Authors:** Lin Chen, Wanliang Shi, Hui Li, Xiuli Sun, Xionglin Fan, Gene LeSage, Hui Li, Yi Li, Yi Zhang, Xiumei Zhang, Ying Zhang, Deling Yin

**Affiliations:** 1 Department of Internal Medicine, College of Medicine, East Tennessee State University, Johnson City, Tennessee, United States of America; 2 Department of Pharmacology, Shandong University School of Medicine, Jinan, People's Republic of China; 3 Department of Molecular Microbiology and Immunology, Bloomberg School of Public Health, Johns Hopkins University, Baltimore, Maryland, United States of America; 4 Department of Obstetrics and Gynecology, Peking University People's Hospital, Beijing, People's Republic of China; University of California Merced, United States of America

## Abstract

**Background:**

Although it is established that opioid and *Mycobacterium tuberculosis* are both public health problems, the mechanisms by which they affect lung functions remain elusive.

**Methodology/Principal Findings:**

We report here that mice subjected to chronic morphine administration and *M. tuberculosis* infection exhibited significant apoptosis in the lung in wild type mice as demonstrated by the terminal deoxynucleotidyl transferase-mediated deoxyuridine triphosphate nick end labeling assay. Morphine and *M. tuberculosis* significantly induced the expression of Toll-like receptor 9 (TLR9), a key mediator of innate immunity and inflammation. Interestingly, deficiency in TLR9 significantly inhibited the morphine and *M. tuberculosis* induced apoptosis in the lung. In addition, chronic morphine treatment and *M. tuberculosis* infection enhanced the levels of cytokines (TNF-α, IL-1β, and IL-6) in wild type mice, but not in TLR9 knockout (KO) mice. The bacterial load was much lower in TLR9 KO mice compared with that in wild type mice following morphine and *M. tuberculosis* treatment. Morphine alone did not alter the bacterial load in either wild type or TLR9 KO mice. Moreover, administration of morphine and *M. tuberculosis* decreased the levels of phosphorylation of Akt and GSK3β in the wild type mice, but not in TLR9 KO mice, suggesting an involvement of Akt/GSK3β in morphine and *M. tuberculosis*-mediated TLR9 signaling. Furthermore, administration of morphine and *M. tuberculosis* caused a dramatic decrease in Bcl-2 level but increase in Bax level in wild type mice, but not in TLR9 KO mice, indicating a role of Bcl-2 family in TLR9-mediated apoptosis in the lung following morphine and *M. tuberculosis* administration.

**Conclusions/Significance:**

These data reveal a role for TLR9 in the immune response to opioids during *M. tuberculosis* infection.

## Introduction

Opioids have been widely applied in clinics as one of the most potent pain relievers for centuries. However, opioids as clinical therapies produce benefits as well as severe side effects. Studies showed that opiates, including morphine, cause cell death and apoptosis in various systems. We have previously reported that opioids promote lymphocyte apoptosis both *in vitro* and *in vivo* [1], [2]. Recent studies have demonstrated that PI3K/Akt signaling is required for opioid induced cell proliferation and the survival signaling pathway with changes in intracellular proapoptotic elements such as Bax, Hsp70 and antiapoptotic elements such as Bcl-2 in response to morphine effects [3]. Apoptotic pathways induced by opioids seem to be mediated by the mitochondrial apoptotic pathway, associated with a decrease in Bcl-2 levels and direct interference with the mitochondrial transmembrane potential [4]. The effects of opioids in various infections are well known [5]. However, very little is known about the effect of opioids on tuberculosis infection.

*M*. *tuberculosis* remains a leading global public health problem despite the availability of chemotherapy and BCG vaccine [6]. A few studies have shown that opioid addicts are more susceptible to mycobacterial infections than non-addicts [7] and can also show anergy towards tuberculin test, thus obscuring the diagnosis of TB [8]. Macrophages infected with *M*. *tuberculosis* undergo increased apoptosis, which is caspase-8 dependent but caspase-9 independent [9].

Toll-like receptors (TLRs) play a critical role in both innate resistance and the initiation of adaptive immunity to infectious agents [10]–[13]. TLRs recognize pathogen-associated molecular patterns (PAMPs) or endogenous inflammation-associated molecules [10], [14], [15]. TLRs recognize distinct molecular structures on microbes, and in several cases (e.g., recognition of viruses by TLR3, TLR7, TLR8, and TLR9), different sets of TLRs have been associated with the response to different classes of microorganisms [10]–[13]. Recent studies have suggested that stimulation of TLRs can result in apoptosis by triggering pro-apoptotic signaling [16]. TLR2 stimulation activates the phosphoinositide 3-kinase (PI3K)/Akt signaling pathway [17]. Akt regulates cellular activation, inflammatory response, and apoptosis. Activated Akt phosphorylates several downstream targets of the PI3K signaling pathway such as GSK3β. GSK3β is a constitutively active enzyme that is inactivated by Akt [17], [18]. GSK3β plays a pivotal role in regulating many cellular functions, including cell survival and apoptosis [17]–[19]. Available evidence suggests that TLR9 participates in the modulation of immune responses to TB [20]. Hoelscher et al. reported that TLR2/4/9-deficient mice controlled *M.* t*uberculosis* replication [21], however, the role of TLR for immune response to *M. tuberculosis* is still controversial [21]. Our studies focus on the role of TLR9 in morphine administration and *M. tuberculosis* infection, not only *M. tuberculosis* infection.

In this study, we show opioids through potent stimulus of TLR9-dependent proinflammatory cytokine production caused altered host resistance against *M. tuberculosis* in the mouse model. In addition, our data demonstrate that mice deficient in TLR9 inhibit morphine-induced apoptosis during *M. tuberculosis* infection. Finally, we found that inhibition of TLR9 prevents morphine and *M. tuberculosis* -induced apoptosis through pro-apoptotic and anti-apoptotic pathways. Taken together, these data reveal a role for TLR9 in the immune response to opioids during *M*. *tuberculosis* infection and provide an important example of TLR9 in host resistance to infection.

## Results and Discussion

### A Deficiency in TLR9 Is Resistant to Morphine and *M. tuberculosis*–Induced Apoptosis

**Figure 1:**
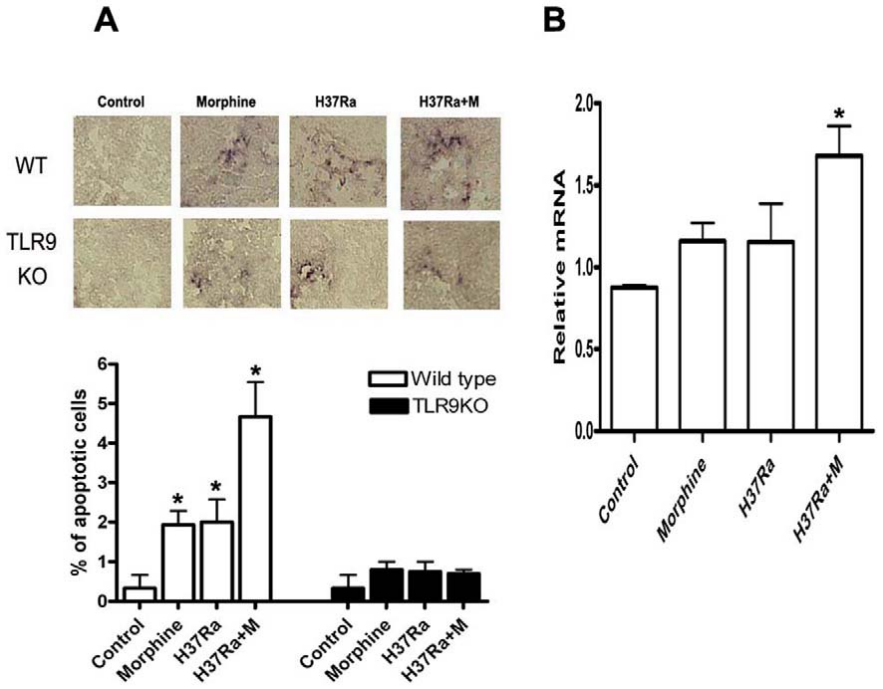
*M. tuberculosis* H37Ra and morphine cause apoptosis. Wild type and TLR9 KO male mice aged 9–10-week were i.p. injected with 2.5×10^7^ H37Ra bacteria in 100 µl. After 4 hours post-injection, mice were implanted subcutaneously with a 25 mg morphine base pellet and the male mice with a placebo pellet implantation as controls as described under Materials and Methods. The lungs were sectioned and apoptotic cells (dark brown color cells) were determined by TUNEL assay (A). Photographs of representative TUNEL-stained cells are shown at the top. Magnification 200×. The bar graph at the bottom shows the percentage of apoptotic cells (A). Results represent mean ± S. E. M. of three independent experiments. The lungs were also harvested for TLR9 expression by real-time quantitative RT-PCR in the wild type mice (B). Means and SEs were calculated from 5–7 mice per group. * *p*<0.01 compared with wild type control group.

We have recently reported that opioids induce lymphocyte apoptosis [1], [2], [22], [23]. We have also shown that opioid promotes cell apoptosis through TLR2 [16]. However, the role of TLRs in opioid-mediated apoptosis during *M*. *tuberculosis* infection is unknown. It has been shown that morphine increase the expression of TLR9 [24]. We therefore asked whether TLR9 is involved in morphine additive effect with *M*. *tuberculosis* infection on inducing apoptosis. During the mouse infection with *M*. *tuberculosis* H37Ra, we implanted wild type mice and TLR9 knockout mice with a 25 mg sustained-release morphine pellet, while control mice received a comparable placebo pellet for different time periods and determined apoptotic cells using the TUNEL technique. We found that chronic morphine administration or H37Ra infection alone induced cell apoptosis in the lungs from wild type mice, but not in TLR9 deficient mice (Fig. 1A). Combination of morphine treatment and H37Ra infection has a significant additive effect on apoptosis in wild type mice. Notably, a deficiency in TLR9 mice is resistant to this additive effect. Therefore, morphine and *M. tuberculosis*-induced cell apoptosis requires TLR9.

We next examined the level of TLR9 in the lungs with or without morphine and H37Ra infection. As shown in Fig. 1 B, the expression of TLR9 in the lungs was significantly enhanced after morphine treatment and H37Ra infection as detected by quantitative real time RT-PCR. These results suggest a prominent role of morphine and H37Ra infection in the induction of TLR9 expression.

### Morphine and H37Ra Induce Inflammatory Cytokine Levels in Wild Type Mice but Not in TLR9 Deficient Mice

**Figure 2:**
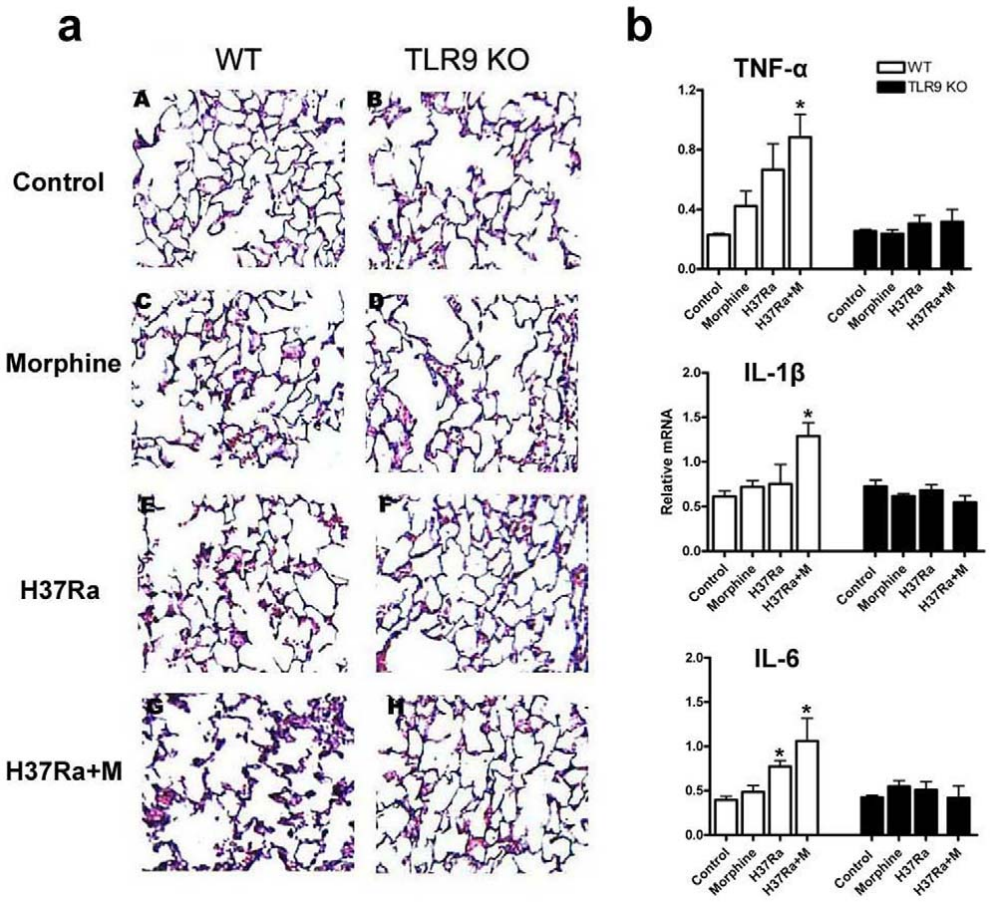
A deficiency in TLR9 is resistant to H37Ra and morphine-induced inflammatory responses. Wild type and TLR9 KO male mice aged 9–10-week were i.p. injected H37Ra and implantation of morphine pellets and placebo pellets as in Fig. 1. (A) The lungs were fixed by formalin, embedded with paraffin, and sectioned (10-µm-thick) and then stained with hematoxylin and eosin. Representative sections from wild type and TLR9 KO mice are shown. Magnification 100×. Data are representatives of at least three independent experiments. (B) The lungs were harvested as Fig. 1 and total RNA was isolated. The expression of TNF-α, IL-1β, and IL-6 was determined by real-time quantitative RT-PCR. The level of gene expression is presented as relative mRNA expression units. Means and SEs were calculated from 5–7 mice per group. * *p*<0.01 compared with wild type control group and H37Ra alone treatment group.

It has been demonstrated that *M*. *tuberculosis* is a potent stimulator of TLR9-dependent pro-inflammatory cytokine production in macrophages [20]. Activation of TLRs stimulates the production of inflammatory cytokines [25]. We determined whether TLR9 plays a role in the inflammatory response following morphine treatment and H37Ra infection. As shown in Fig. 2A, either morphine or H37Ra alone induced inflammation with hyperaemia in the lung in wild type mice, but not in TLR9 knockout mice. Of greater significance, a deficiency of TLR9 in mice significantly attenuated morphine and H37Ra-induced inflammation with hyperaemia.

We then examined the expression levels of proinflammatory cytokines in TLR9 KO and wild type mice following morphine administration and H37Ra infection. Administration of morphine and *M. tuberculosis* dramatically increased the expression of TNF-α, IL-1β, and IL-6 in the wild type mice. Interestingly, significantly (2- to 3-fold) lower expression of IL-1β, and IL-6 was observed in the TLR9 KO mice as compared with the wild type control mice (Fig. 2B). These data suggest that a pro-inflammatory role for TLR9 in morphine treatment and H37Ra-mediated immune responses.

### TLR9 Deficiency Prevents Morphine and *M. tuberculosis* H37Ra-Decreased Phosphorylation of Akt and GSK3β

**Figure 3:**
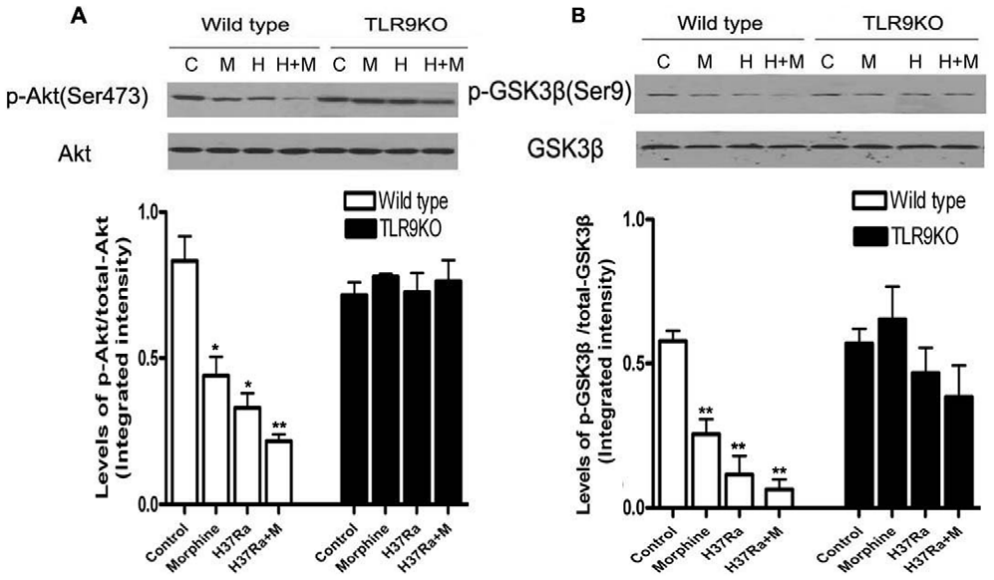
Role of TLR9 in H37Ra and morphine-mediated Akt and GSK-3β phosphorylation. The lungs were harvested from wild type and TLR9 KO mice after H37Ra and morphine administration as in Fig. 1. Total and p-Akt (A), total and p-GSK3β (B) were determined by Western blot. Representative results of the level of p-Akt, total Akt, p-GSK3β and p-GSK3β are shown at the top of each pane. Means and SEs were calculated from 5 mice per group. * *p*<0.01 compared with wild type control group.

Growing evidence suggests that there is a cross-talk between TLR signaling and the PI3K/Akt signaling pathway [17], [18]. Additionally, the PI3K/Akt signaling pathway may be an endogenous negative feedback regulator of TLR4-mediated immune responses. Our previous studies showed that TLR2-mediated morphine induced apoptosis through PI3K/Akt/GSK3β in neurons. In the present study, to examine whether TLR9 can activate Akt signaling following morphine and H37Ra infection in wild type mice and TLR9 KO mice, we determined the levels of phosphorylated Akt (phospho-Akt) at Ser^473^. The levels of phospho-Akt at Ser^473^ in the lungs from wild type mice were significantly lower compared with control (without treatment) wild type mice (Fig. 3A). Importantly, reduced levels of phospho-Akt at Ser^473^ caused by morphine and H37Ra were inhibited in the lung from TLR9 KO mice (Fig. 3B). These results suggest that morphine and H37Ra decrease Akt activity through a TLR9-dependent mechanism.

GSK3β is an important downstream target of the Akt signaling pathway [19]. Phosphorylation of GSK-3β on the inactivating residue serine-9 by Akt results in GSK-3β inactivation [17], [19]. We investigated the effect of TLR9 on phosphorylation of GSK-3β with or without morphine treatment and H37Ra infection. As shown in Fig. 3B, morphine and H37Ra significantly decreased the levels of GSK3β serine-9 phosphorylation in the lungs from the wild type mice, but not from TLR9 KO mice. Since phosphorylation of serine-9 is inhibitory for GSK3β activity, these data demonstrate that morphine and H37Ra decrease GSK3β activity through a TLR9-dependent signaling pathway.

### Effect of TLR9 on the Levels of Bcl-2 and Bax following Morphine Administration and H37Ra Infection

**Figure 4:**
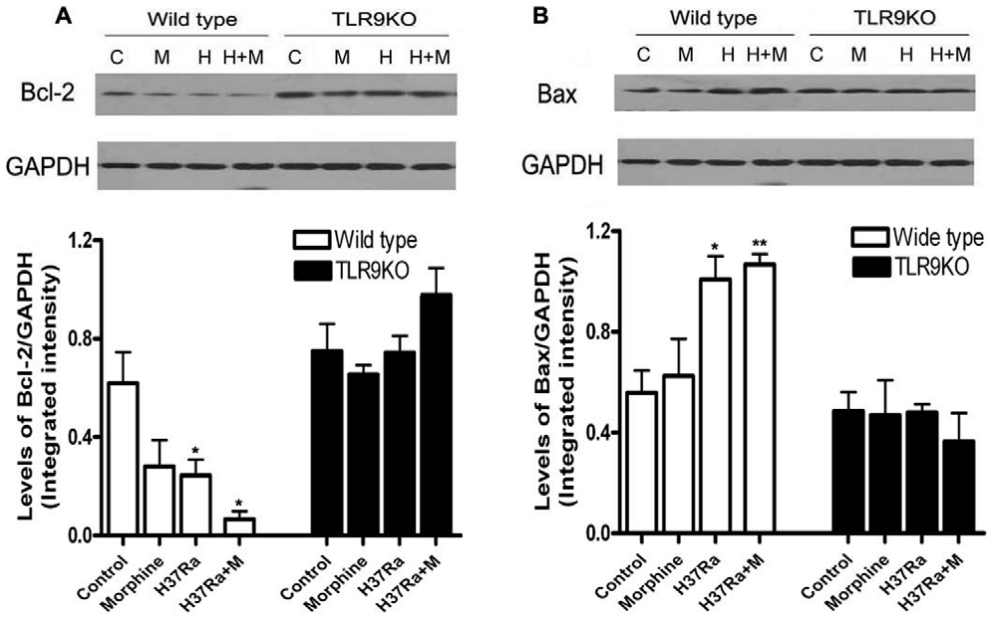
Effect of TLR9 in H37Ra and morphine-mediated expression of Bcl-2 and Bax. We harvested lungs from wild type and TLR9 KO mice following H37Ra and morphine administration as Fig. 1. The expression of Bcl-2 (A) and Bax (B) was examined by Western blot analysis. Representative results of the levels of Bcl-2 and Bax are shown at the top of each pane. Means and SEs were calculated from 5 mice per group. * *p*<0.01 compared with control wild type group.

Activated Akt induces the alteration of Bcl2 and Bax expression and plays an antiapoptotic role in several different cell types [26]. It has been demonstrated that TLR9 agonist CpG-DNA induces phosphorylation of Bad, a pro-apoptotic member of the Bcl-2 family, at Ser^112^ and Ser^136^ through activation of Akt [27]. Phosphorylated Bad then dissociates from Mcl-1, allowing the expression of anti-apoptotic effects [28]. We determined the expression of pro-apoptotic protein Bax and anti-apoptotic protein Bcl-2 after treatment of morphine and H37Ra infection. Treatment of morphine with R37Ra infection significantly decreased the levels of Bcl-2 (Fig. 4A) and increased the levels of Bax (Fig. 4B) in the lung from wild type mice. However, morphine with H37Ra did not alter the levels of Bcl-2 (Fig. 4A) and Bax (Fig. 4B) in the lung from TLR9 KO mice. Taken together, our results suggest that Bcl-2 family is involved in TLR9-mediated apoptosis in the lung following morphine and H37Ra administration.

### Deficiency in TLR9 Attenuates Morphine and H37Ra-Induced Increase in Lung CFU Counts

**Figure 5:**
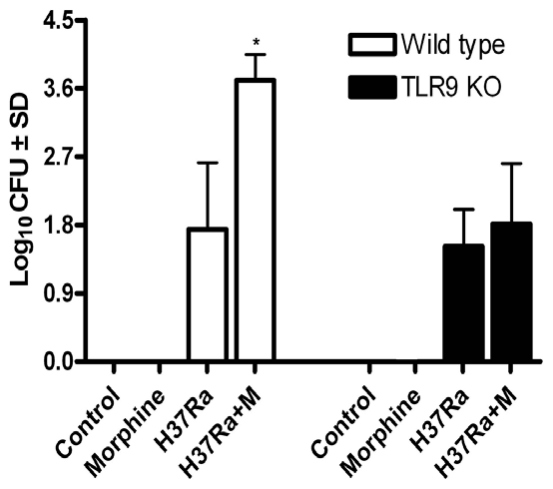
A deficiency in TLR9 is resistant to H37Ra and morphine-induced lung CFU values. After H37Ra and morphine administration, the lungs were harvested from wild type and TLR9 KO mice and CFU counts were determined as described under Materials and Methods. Data are from three independent experiments. Means and SEs were calculated from 5 to 7 mice per group. * *p*<0.01 compared with control wild type group.

To examine the effects of TLR9 on host resistance manifested by changes in pulmonary bacterial load after morphine administration and H37Ra infection, TLR9 KO mice and wild type mice were chronically administered with morphine during H37Ra infection. The CFU count in the lungs was determined by plating the lung homogenates on 7H11 agar plates. As shown in Fig. 5, significantly greater CFU count was observed in the lung tissues when wild type mice were treated with both morphine and H37Ra than they were treated with either morphine or H37Ra alone. Notably, morphine and H37Ra-indcued increase in CFU count was significantly inhibited in the lung tissues from the TLR9 KO mice, suggesting that this additive effect requires TLR9. Singh et al. [29] have suggested that morphine exerts a dose-dependent effect in murine tuberculosis. Our data demonstrate a role of TLR9 in the immune responses to opioids during *M*. *tuberculosis* infection and provide an important example of TLR9 in host resistance to infection.

The studies presented here show that TLR9 deficient mice exhibit distinct defects in immune response to opioids and *M. tuberculosis in vivo*. Moreover, as discussed above, in several cases individual immune deficiencies appeared to be linked to TLR9. For example, morphine and H37Ra-induced TNF, IL-1, and IL-6 production appears to be controlled by TLR9 (Fig. 2). The findings of this study have several implications for the investigation of the role of TLRs in susceptibility of humans to drug abuse and mycobacterial infections. The involvement of these receptors has been suggested from genetic studies correlating single nucleotide polymorphisms within the TLR9 gene, with disease susceptibility to infection in populations exposed to *M. tuberculosis* [30]. A limitation of the study is the use of attenuated strain *M. tuberculosis* H37Ra. Virulent strains such as *M. tuberculosis* H37Rv will be used in future studies.

An outbreak of multidrug-resistant tuberculosis at a methadone treatment program has been identified recently [31], which underscores the emergency of developing effective strategies to prevent TB transmission in such setting. Our findings presented here demonstrate a critical role of TLR9 involved in the chronic morphine administration induced impairment of pulmonary immune response to murine tuberculosis infection. TLR9 is known to recognize oligonucleotide derived from *M. tuberculosis* genome [31]. Further studies are needed to determine the mechanisms by which opioid administration may affect the immune system and the susceptibility to mycobacterial infections.

## Materials and Methods

### Experimental Animals

TLR9 KO mice on a Balb/c background were kindly provided by Dr. Shizuo Akira (Osaka University, Osaka, Japan) via Dr. Dennis Klinman (National Cancer Institute, Frederick, MD). Wild type Balb/c mice were purchased from the Jackson Laboratory (Bar Harbor, ME) and maintained in the Division of Laboratory Animal Resources at East Tennessee State University (ETSU), a facility accredited by the Association for the Assessment and Accreditation of Laboratory Animal Care International (AAALAC). All aspects of the animal care and experimental protocols were approved by the ETSU Committee on Animal Care. Male mice aged 8 weeks were used in all experiments.

### Reagents

Placebo pellets and morphine pellets were kindly provided by National Institutes of Drug Abuse (NIDA). The antibodies to total Akt, phosphor-Akt (Ser 473), total GSK3β, phospho-GSK3β (Ser9) were purchased from Cell Signaling Technology (Beverly, MA). The antibodies to Bcl-2, Bax, and GAPDH were purchased from Santa Cruz Biotechnology (Santa Cruz, CA). In situ apoptosis detection kit was purchased from Roche Diagnostic (Indianapolis, IN). The Quantitative PCR kit was purchased from Invitrogen (Carlsbad, CA). *M. tuberculosis* strain H37Ra was grown in 7H9 liquid medium (Difco) supplemented with 0.05% Tween 80 and 10% BSA-dextrose-catalase (ADC) enrichment (Difco) at 37°C for approximately 2 weeks with occasional agitation.

### Experimental Protocol

Wide type and TLR9 KO mice were randomly divided into four groups of 10 each respectively: control group (C), morphine group (M), H37Ra group (H37Ra), and H37Ra with morphine group (H37Ra + M). Each of the mice in H37Ra and M groups received an intraperitoneal (i.p.) injection with 2.5×10^7^ bacteria in 100 µl [29]. Each of the mice in C and M groups were i.p. injected with 100 µl PBS. After 4 hours injection, mice in M and H37Ra with M group were anesthetized with inhaled isofluorane and implanted subcutaneously with a 25 mg morphine base pellet and the male mice with a placebo pellet implantation served as controls. Under isoflurane anesthesia, a small skin pocket was dissected in the animal's back, where a single pellet was inserted and the skin was closed with surgical sutures [32]–[34]. Fifteen days later, the second implantations were performed. Another fifteen days after second implantation, mice were sacrificed and the lungs were harvested. Partial lungs were excised and frozen in liquid nitrogen for CFU enumeration, gene and protein analysis. Partial lungs were submerged in 4% paraformaldehyde in PBS, fixed with formalin and embedded with paraffin. Other lungs were immediately frozen by liquid nitrogen for molecular and cellular studies.

### CFU Enumeration

The lungs were homogenized in 1 ml PBS buffer containing 0.1% Tween 80. The suspension was diluted in serial 10-fold dilutions. Triplicate samples (50 µl) were plated on 7H11 plates supplemented with ADC, and antibiotic cocktail (cycloheximide 10 µg/ml, carbenicillin 50 µg/ml, polymyxin B 25 µg/ml and trimethoprim 20 µg/ml) to prevent contamination. The CFU values were counted after 3 week incubation at 37°C.

### Measurement of Cytokine Gene Expression in Lung Tissues

Total RNA was isolated from lungs by the VERSA GENE™ RNA Tissue Kit (Gentra SYSTEMS; Minnesota) and the real-time RT-PCR detection technique was performed as described previously [23]. Briefly, first-strand cDNA was synthesized from 1 µg of total RNA using a Reaction Ready™ first strand cDNA synthesis kit (SABioscience Corporation, Frederick, MD). After incubation at 70°C for 3 min and cooling down to 37°C for 10 min, RT cocktail was added to the annealing mixture and further incubated at 37°C for 60 min. 2 µl of 1∶2 diluted cDNA was subjected to real-time quantitative PCR using Bio-Rad iCycler iQ Multicolor Real-Time PCR Detection System (Bio-Rad Life Science Research, Hercules, CA). PCR was performed in a 25 µl volume using SYBR GreenER qPCR Super Mix for iCycler (Invitrogen Corporation, Carlsland, CA). All primers were purchased from SuperArray Bioscience Corporation. All PCR assays were performed in triplicate. The following primer pairs were used: for TLR9: CCCTGGTGTGGAACATCAT(forward) and GTTGGACAGGTGGACGAAGT (reverse); TNF-α: AGTTCCCAAATGGCCTCCCTCTCA(forward) and TGGTTTGCTACGA CG-TGGGCTACA(reverse); IL-1β: CTGGAGAGTGTGGATCCCAAGCAA(forward) and GGGAACTC- TGCAGACTCAAACTCCAC(reverse); IL-6: AGCCAGAGTCCTTCAGAGAGATACAG(forward) and CTCCAGCTTATCTGTTAGGAGAGCA(reverse).

### Detection of Apoptosis by TUNEL Assay

The frozen sections from wild type and TLR9 KO mice were harvested for terminal deoxynucleotidyl transferase biotin-dUTP nick end labeling (TUNEL) assay. Nucleosomal DNA fragmentation in cells was determined by the TUNEL assay using an in situ apoptosis detection kit according to the manufacturer's instructions and our previous publications [22], [35]. The number of apoptotic cells was counted in randomly selected fields to calculate the ratio of apoptotic cells and total 500 cells.

### Histopathology

Lungs were fixed by formalin, embedded with paraffin, sectioned and then stained with hematoxylin and eosin.

### Western Blot Analysis

Western blotting was performed as described in our previous publications [35], [36]. Briefly, protein concentration was measured using the bicinchoninic acid kit according to the manufacturer's instructions. Equal amounts of protein were separated by 12% SDS-PAGE then transferred to a nitrocellulose membrane (Bio-Rad, Hercules, CA). The membrane was then incubated at room temperature in a blocking solution composed of 5% skim milk powder dissolved in 1×TBS for 1 h. The membrane was then incubated overnight at 4°C with the blocking solution containing first antibody. After washing three times with TBS-T for 5 min, the blot was incubated with a second antibody. The blot was again washed three times with TBS-T before being exposed to the SuperSignal West Dura Extended Duration substrate (Pierce Biotechnology, Rockford, IL).

### Statistical Analysis

All data were represented as means ± SEM. Differences among groups were compared by one-way analysis of variance (ANOVA) followed by Tukey's multiple-comparison test. *P*<0.05 was considered to be significant.

## Acknowledgments

The authors wish to express their appreciation to Dr. Shizuo Akira (Osaka University, Osaka, Japan) and Dr. Dennis Klinman (National Cancer Institute, Frederick, MD), for providing TLR9 knockout mice. We thank NIDA for providing us with both placebo and morphine pellets.
